# Identification of Potential Biomarkers for CAD Using Integrated Expression and Methylation Data

**DOI:** 10.3389/fgene.2020.00778

**Published:** 2020-09-09

**Authors:** Xiaokang Zhang, Yang Xiang, Dingdong He, Bin Liang, Chen Wang, Jing Luo, Fang Zheng

**Affiliations:** Department of Clinical Laboratory Medicine and Center for Gene Diagnosis, Zhongnan Hospital of Wuhan University, Wuhan, China

**Keywords:** coronary artery disease, methylation, FN1, PTEN, POLR3A

## Abstract

DNA methylation plays an essential role in the pathogenesis of coronary artery disease (CAD) through regulating mRNA expressions. This study aimed to identify hub genes regulated by DNA methylation as biomarkers of CAD. Gene expression and methylation datasets of peripheral blood leukocytes (PBLs) of CAD were downloaded from the Gene Expression Omnibus (GEO) database. Subsequently, multiple computational approaches were performed to analyze the regulatory networks and to recognize hub genes. Finally, top hub genes were verified in a case-control study, based on their differential expressions and methylation levels between CAD cases and controls. In total, 535 differentially expressed-methylated genes (DEMGs) were identified and partitioned into 4 subgroups. TSS200 and 5′UTR were confirmed as high enrichment areas of differentially methylated CpGs sites (DMCs). The function of DEMGs is enriched in processes of histone H3-K27 methylation, regulation of post-transcription and DNA-directed RNA polymerase activity. Pathway enrichment showed DEMGs participated in the VEGF signaling pathway, adipocytokine signaling pathway, and PI3K-Akt signaling pathway. Besides, expressions of hub genes fibronectin 1 (FN1), phosphatase (PTEN), and tensin homolog and RNA polymerase III subunit A (POLR3A) were discordantly expressed between CAD patients and controls and related with DNA methylation levels. In conclusion, our study identified the potential biomarkers of PBLs for CAD, in which FN1, PTEN, and POLR3A were confirmed.

## Introduction

Coronary artery disease (CAD), as the main type of cardiovascular disease, has become one of the leading causes of morbidity and mortality in both developed and developing countries (Wood and Eisele, 2017). This acute tendency is due to the population aging. According to the CAD prediction model, in China, more than 20 million deaths and 16 million instances of labor loss will be attributed to CAD from 2000 to 2029 (Moran et al., 2008). The total attributed to the social economy connected with CAD in developing countries was estimated to be approximately 3.7 trillion dollars in 2010, which is roughly equal to 1–3% of Gross Domestic Product (GDP) across developing countries (Gaziano et al., 2017).

Coronary arteriography (CAG) is the gold standard of CAD diagnosis, but the high cost and invasiveness limit its application (De Marco et al., 2018), whereas the cheaper cost and less invasive nature of blood biomarker detection make it easier to promote (Rusnak et al., 2017). Epigenetics is defined as the heritable transcriptional modifications that are not induced by the nucleotide sequence alterations of DNA (Duan et al., 2018). Multiple factors such as environment, diet, oxidative stress, and inflammatory stimuli influence epigenetic contents, including DNA methylation, RNA methylation, chromatin histone modification, non-coding RNA and DNA methylation, among which DNA methylation is the most indagated (Xu et al., 2019). For example, prolonged hypoxia can induce epigenetic modifications in myocardial fibroblasts, since methylation status of the genome and specific genes are affected by DNA methyltransferase (DNMT), which is regulated by a hypoxia inducible factor 1α (HIF-1α) (Watson et al., 2014). While studies have shown that in women who lost weight by lowering their calorie intake, the DNA methylation levels of Leptin and TNF-α promoters were significantly reverse modified, and the risk of CAD was significantly reduced (Cordero et al., 2011).

Aberrant DNA methylation participates in various processes of CAD development by regulating the mRNA expression of interrelated genes. For instance, ABCA1 plays an essential part in reverse cholesterol transport (RCT) by combining with apoA-I to form high-density lipoprotein (HDL) in the cell membrane and promoting the excretion of free cholesterol and phospholipids from cells. ABCA1 weakens the chemotactic ability of macrophages by reducing the content of free cholesterol in the cell membrane and delays the pathological progress of CAD (Bashore et al., 2019). The demethylation of the ABCA1 promotor has been verified to be related to the high expression of ABCA1, which can accelerate the process of CAD by expediting the formation of foam cells and thrombogenesis (Peng et al., 2014; Ghaznavi et al., 2018). Intriguingly, there is a conspicuous correlation between the methylation status of the ABCA1 promoter and physiological age. The ABCA1 promoter is hypermethylated in aged CAD patients, which can be partially illustrated by the accumulation of aberrant epigenetic changes during the long-term disease states (Ghaznavi et al., 2018). Cystathionine gamma-lyase, encoded by *CTH*, is a crucial part of the homocysteine metabolism pathway (Szijarto et al., 2018). Previous studies have found that hypermethylation of the *CTH* promotor in hyperhomocysteinemia in mice can lead to the decrease of *CTH* expression, which in turn prevents homocysteine from being catabolized and causes vascular endothelial cells injury, eventually results in CAD (Li et al., 2015; Giannakopoulou et al., 2017). A similar phenomenon has been observed in male CAD patients, while the methylation level of *CTH* promotor in female patients is not different from normal controls (Latini et al., 2004). Aberrant methylation status of the promoter has also been proved to impact the inflammatory pathways, which are well known to participate in the progress of CAD by regulating the number, ratio, and function of immune cells. PTX3 accelerates the formation of atherosclerotic plaques by enhancing the migration and chemotactic ability of macrophages, promotes vascular endothelial damage, and exacerbates vascular inflammation. The methylation level of the PTX3 promoter in CAD patients is much lower compared with controls, while higher PTX3 concentration and neutrophil to lymphocyte ratio (NLR) are detected in CAD patients. It indicates that the methylation level of the PTX3 promoter impacts the expression of PTX3 and regulates the number and classification ratio of white blood cells, aggravates an inflammatory response, and then participates in the progress of CAD (Guo et al., 2016).

However, in the past a few years, research on DNA methylation has mainly focused on the connection between methylation conditions of promoter regions and the expression of genes. Recently the aberrant methylation status of other gene regions has also been identified to be associated with CAD, but these complex regulatory networks remain largely unexplored (Oudejans et al., 2016; Nakatochi et al., 2017; Yamada et al., 2018). Therefore, an integrative research study was required, combining both genomic expression profile and epigenomic DNA methylation of PBLs in CAD in Chinese populations. In our study, we calculated the methylation status of 5′-C-phosphate-G-3′ (CpGs) sites in different intragenic gene regions, including TSS1500, TSS200, 5′UTR, 1stExon, body, and 3′UTR. Besides, we consolidated DNA methylation and mRNA expression data to recognize genes functioning in CAD and regulated by DNA methylation, which might be potential PBLs biomarkers. We identified hub genes that were both aberrantly methylated and differentially expressed in CAD patients compared with controls. Vital hub genes were validated in a case-control study to enhance the reliability of bioinformatics analysis. Based on the combined results of bioinformatics analysis and clinical sample validation, we aimed to ascertain novel feasible PBLs biomarkers and shed light on their possible roles in the pathogenesis of CAD.

## Materials and Methods

The methods used in our study mainly contained microarray data collection, differential expression, and methylation analysis, functional and pathway enrichment analysis, Protein-protein interaction (PPI) network establishment, module analysis, and hub genes identification, followed by experimental validation in PBLs, correlation analysis, and multivariate stepwise linear regression analysis. The research flow diagram of this study is shown in Supplementary Figure S1.

### Microarray Data Collection

We retrieved GEO of The National Center for Biotechnology Information (NCBI) to screen datasets that contained profiling information about mRNA expressions and DNA methylations in CAD patients versus controls. A series of datasets were obtained and only those that met both the inclusion and exclusion criteria were analyzed. The detailed inclusion criteria were as follows: (Wood and Eisele, 2017) datasets involved mRNA expression information or DNA methylation status detected from PBLs; (Moran et al., 2008) those that contained both CAD patients and controls; (Gaziano et al., 2017) sample size was no less than 5 of each subgroup. Besides, datasets were excluded if the specimen type was one of the components of PBLs, such as monocytes, granulocytes, or platelets. Only two datasets were up to the selection criteria, GSE42148, and GSE107143. Gene expression profiling array (GSE42148), measured by the Agilent-028004 SurePrint G3 Human GE 8 × 60K Microarray, provided mRNA expression data from 11 controls with normal electrocardiogram diagnoses and 13 CAD patients. The series matrix and platform files (GPL13607) were downloaded from the GEO database. The genome-wide DNA methylation profiling array (GSE107143) contained information on DNA methylation status from 8 controls with normal physical conditions and 8 CAD patients. The data were measured by Illumina HumanMethylation450 BeadChip and the series matrix file, as well as the platform file (GPL13534), which were obtained from the GEO database. In consideration of mRNA expression array, GSE71226 did not meet the inclusion criteria with a small sample size of 3 CAD patients and 3 controls, meaning we only used it to evaluate the discriminating ability of candidate gene mining.

### Differential Expression Analysis

The R package named “limma” was utilized to select differentially expressed genes (DEGs) from the series matrix file downloaded from the GEO database (Ritchie et al., 2015). Probes without matching gene symbols were deleted and genes with multiple probes were averaged in the subsequent analysis. We took *P* < 0.05 and absolute value of log_2_FC (fold change) > 0.3 as the threshold of significant DEGs. A heatmap based on the expression data was drawn using the R package “pheatmap.”

### Differential DNA Methylation Analysis

AS, one of the mainstream detection platforms for DNA methylation, Illumina HumanMethylation450 BeadChip covered roughly 450,000 CpGs that randomly separate in different gene regions, including TSS1500, TSS200, 5′UTR, 1stExon, body, 3′UTR, and intergenic regions. TSS1500 and TSS200 are regions from 201 to 1500 bases and 1 to 200 bases of the upstream of transcriptional start site (TSS), respectively. The “5′UTR (5′ untranslated region)” is considered as the region between TSS and the first initiation codon. “1stExon (the first exon)” is one of the most extensively studied translated regions that is generally influenced by methylation status. “Body” stands for the sequence from the first initiation codon to the stop codon of a gene. The “3′UTR (3′ untranslated region)” is the area between the stop codon and poly-A tail. The 6 intragenic regions mentioned above are the main components of a gene and we took the average of the beta value of CpGs from the same region as the comprehensive methylation level of each intragenic region. The limma package of R was used for identification of differentially methylated CpGs sites (DMCs), differentially methylated regions (DMRs), and differentially methylated genes (DMGs) with the threshold *P* < 0.05 and log_2_FC > 0.3. Single CpGs met the threshold were taken as DMCs, meanwhile, intragenic regions that matched the threshold were identified as DMRs. Genes with one or more DMRs that differentially methylated in the same direction were considered as DMGs. We defined genes that were identified both as DEGs and DMGs as differentially expressed-methylated genes (DEMGs). The Upset plot performed by R package “UpSetR” was utilized to describe the distribution of DMCs in different intragenic regions (Conway et al., 2017). The locations of DMCs on chromosomes were visualized by R package “RIdeogram” (Zhaodong et al., 2019).

### Functional and Pathway Enrichment Analysis

The r package “clusterProfiler” was USED to implement Gene ontology (GO) enrichment analysis and the Kyoto Encyclopedia of Genes and Genomes (KEGG) for pathway analysis (Kanehisa and Goto, 2000; Gene Ontology Consortium, 2006). More precisely, GO enrichment analysis was carried out within 3 classical subschemas: biological process (BP), cellular component (CC), and molecular function (MF). Subsequently, we utilized “ggplot2” for visualization of the results. The cutoff value of statistical significance was set as *P* < 0.05.

### PPI Network Establishment, a Module Analysis, and Hub Gene Identification

A PPI network was preliminarily constructed through the Search Tool for the Retrieval of Interacting Genes (STRING) database, as a way to explore the inherent relation and regularity of DEMGs. The cutoff value of the interaction score in the STRING database was set at 0.4. To make the PPI network more legible, we used Cytoscape to visualize the network based on interaction information calculated from STRING (Shannon et al., 2003). An auxiliary application named Molecular Complex Detection (MCODE) from Cytoscape was used for module analysis to identify modules with significant interaction under threshold MCODE scores > 3, k-score = 2 and nodes numbers > 4. CytoHubba, another application from Cytoscape, provided 12 algorithms to estimate evidence levels of interaction within genes from the PPI network (Chin et al., 2014). We summarized these 12 evaluation scores as the comprehensive assessment standard for screening top hub genes.

### Study Population and PBLs Collection

We performed a case-control study to consolidate the expression status of hub genes filtered through bioinformatics analysis. PBLs of 40 CAD patients from Zhongnan Hospital of Wuhan University (Wuhan, China) were collected from December 2018 and July 2019. The diagnostic criterion for CAD was based on coronary angiography that showed stenosis caused by atherosclerotic plaque was more than 50% in at least one coronary artery. Meanwhile, 36 age and sex matched people who were negative in the examination of ultrasound or coronary CTA or coronary angiography were enrolled as controls. None of the participants were diagnosed with the following diseases: cancer, acute inflammation, hematological system disorders, congenital heart disease, history of previous myocardial infarction (MI), hepatic failure, or other severe disorders. The basic information and clinical characteristics of participants are shown in Table 1. Our study was authorized by the Medical Ethics Committee of Zhongnan Hospital of Wuhan University.

**TABLE 1:** Clinical characteristics of subjects in validation study.

Characteristic	Controls	CAD patients	*P* value
**Demographics**			
Male/Female	17/13	22/8	0.2789
Age (year)	55.27 ± 9.03	59.80 ± 9.26	0.0598
Risk factors			
**History of HP (yes/no)**	9/21	20/10	**0.0092**
**History of DM (yes/no)**	1/29	8/22	**0.0257**
**Clinical parameters**			
TC (mmol/L)	4.25 ± 0.72	4.53 ± 1.45	0.3429
**TG (mmol/L)**	1.14 ± 0.39	1.89 ± 1.43	**<0.001**
LDL-C (mmol/L)	2.60 ± 0.60	2.80 ± 1.08	0.3701
HDL-C (mmol/L)	1.41 ± 0.34	1.30 ± 0.47	0.2964
FPG (mmol/L)	5.48 (5.03, 5.73)	5.69 (5.18, 6.90)	0.0740
WBC (× 10^9^)	5.84 ± 1.33	6.33 ± 1.77	0.2290
**Monocyte (× 10^9^)**	0.43 ± 0.11	0.58 ± 0.21	**<0.001**
**Neutrophil (× 10^9^)**	3.31 ± 1.03	3.97 ± 1.28	**0.0315**
**Lymphocyte (× 10^9^)**	1.93 ± 0.51	1.60 ± 0.61	**0.0250**
**LMR (ratio)**	4.68 ± 1.17	2.98 ± 1.21	**<0.001**
NMR (ratio)	7.70 (6.13, 8.85)	6.48 (5.53, 8.90)	0.1973
**NLR (ratio)**	1.68 (1.30, 2.05)	2.58 (1.94, 3.20)	**<0.001**

*Data were showed as mean ± SD, median (25 percentiles, 75 percentiles). HP, hypertension; DM, diabetes mellitus; TC, total cholesterol; TG, triglycerides; LDL-C, low-density lipoprotein cholesterol; HDL-C, high-density lipoprotein cholesterol; FPG, fasting plasma glucose; WBC, white blood cell; LMR, lymphocyte to monocyte ratio; NMR, neutrophil to monocyte ratio; NLR, neutrophil to lymphocyte ratio. Entries in bold font indicate statistically significant (*P* < 0.05).*

### The mRNA Expression Analysis

RNA was isolated from PBLs of 30 CAD patients and 30 controls using TRIZOL reagent (Life Technologies, United States). To assess the concentration and purity of RNA, NanoDrop 2000C was applied. About 1 microgram RNA of each sample was used for reverse transcription into complementary DNA (cDNA) through the PrimeScript^TM^ RT reagent kit with gDNA Remover (Takara, Japan). The qPCR was carried out using SYBR Green I UltraSYBR Mixture (CWBIO, China) on Bio-Rad CFX96 (Bio-Rad Laboratories, United States). We took *glyceraldehyde 3-phosphate dehydrogenase (GAPDH)* as an endogenous reference gene to normalize the expression level among multiple samples. The specific sequences of each pair of primers were available in Supplementary Table S1. All experiments were performed twice. Relative gene expression status was calculated by the 2^–ΔCq^ method, in which ΔCq stands for the difference between the mean Cq (quantification cycle) of a target gene and the endogenous reference gene (*GAPDH*).

### The DNA Methylation Analysis

Genomic DNA was extracted from the PBLs of 30 CAD patients and 30 controls using standard phenol/chloroform extraction. DNA was quantified by the NanoDrop-2000C (Thermo Fisher Scientific, United States) and stored at -20°C until use. Due to the limited volume of the PBLs, PBLs from 10 CAD patients and 6 controls were only utilized to extract DNA, and PBLs from 10 CAD patients and 6 controls were only used to extract RNA. PBLs from 20 CAD patients and 24 controls were used to extract both DNA and RNA. Methylation-dependent restriction enzyme digestion based quantitative PCR (MDRE-qPCR) and methylation-sensitive restriction enzyme digestion based quantitative PCR (MSRE-qPCR) were adopted in methylation detection (Redshaw et al., 2014). Methylation-dependent restriction enzyme MspJI and FspEI (New England Biolabs, United States) were used in the analysis of FN1 and PTEN, respectively. POLR3A was detected using methylation-sensitive restriction enzyme Hin6I (SibEnzyme, China). The sequences of primers used in the experiments were listed in Supplementary Table S1. The methylation level was calculated by 100% × [1–2^ΔCq (undigested–digested)^] in MDRE-qPCR, while 100% × 2^ΔCq (undigested–digested)^ was the formula used in MSRE-qPCR (Zhang et al., 2015). To verify the efficacy of enzyme digestion, Methylated HCT116 gDNA, and Unmethylated HCT116 DKO gDNA (Takara, China) was adopted as the positive control and the negative control, respectively, in each experiment. Only when the methylation levels of positive controls were close to 1 and the methylation levels of negative controls were close to zero, the enzyme digestion could be taken as eligible. All experiments were performed twice to enhance the dependability.

### Statistical Analysis

Mean ± standard deviation (SD) was utilized to describe the basic information and clinical characteristics that were normal distributed continuous variables. Abnormal distributed continuous variables were depicted as the median and inter-quartile range. Categorical variables were exhibited by frequencies. We applied a student’s *t* test or Mann-Whitney *U* tests to compare the difference between 2 groups based on the distribution type. Chi-square test or Fisher’s exact test were performed, enabling comparison of categorical variables between groups. The Pearson or Spearman test was used for correlation analysis. We utilized multivariate stepwise linear regression to eliminate interference factors in regression analysis. The receiver operation curve (ROC) was drawn to appraise the diagnostic value of hub genes. Youden’s index was used to screen out the optimum cutoff point of sensitivity (Se) and specificity (Sp). All statistical analyses of this research were conducted through SPSS version 25.0 (SPSS Inc., United States) and GraphPad Prism 8.0 (GraphPad Inc., United States). A statistically significant threshold of two-sided *P* value was set at 0.05.

## Results

### General Characteristics of DEGs, DMCs, DMGs, and DEMGs

**FIGURE 1:**
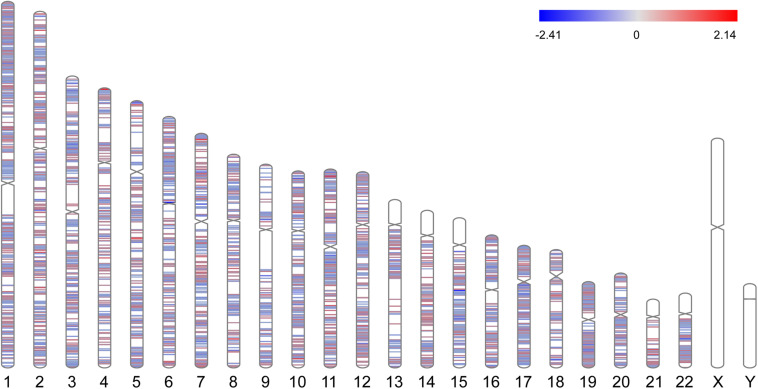
Chromosome distributions of DMCs. Hypermethylated DMCs were marked in red, hypomethylated DMCs were marked in blue.

A total of 3351 DEGs were identified, among which 1863 genes were up-regulated, and 1488 genes were down-regulated in CAD patients’ PBLs compared with controls’. Another microarray dataset from GEO (GSE107143) was used to explore DMCs among approximately 450, 000 CpGs in 8 CAD patients and 8 controls. In aggregate, 7694 DMCs were identified and 3362 of DMCs were hypermethylated, and the other 4332 DMCs were hypomethylated according to the log_2_FC of delta of beta value. The distribution of DMCs on chromosomes is exhibited in Figure 1. Interestingly, none of DMCs were found on sex chromosomes nor the short arms of chromosomes 13, 14, 15, 21, and 22. Meanwhile, it could be observed that DMCs in regions around centromeres were relatively sparse compared to other chromosome regions. To further investigate whether the difference in DMCs density distribution was significant statistically, we calculated the DMCs density in centromere regions and other chromosome regions. We took the 11.1 subbands from both the short arm and the long arm of one chromosome as the centromere region based on genome version GRCh37.p13. As shown in Table 2, the DMCs density was much lower in centromere when compared with other regions (*P* < 0.001), which indicates that there might be a correlation between chromosome regions and gene methylation status.

**TABLE 2:** Density distribution of intragenic DMCs in centromere and other chromosome regions.

Chromosome ID	Centromere length (Mb)	Other regions length (Mb)	DMCs in centromere	DMCs in other regions	DMCs density in centromere (n/Mb)	DMCs density in other regions (n/Mb)	*P* value
1	7.40	241.85	0	805	0.00	3.33	<0.001*
2	6.30	236.68	1	486	0.16	2.05	
3	6.00	192.02	2	465	0.33	2.42	
4	4.50	186.65	1	294	0.22	1.58	
5	4.60	176.32	0	445	0.00	2.52	
6	4.60	166.52	0	607	0.00	3.65	
7	3.70	155.44	0	440	0.00	2.83	
8	5.00	141.36	1	257	0.20	1.82	
9	3.40	137.81	0	172	0.00	1.25	
10	4.30	131.23	2	355	0.47	2.71	
11	4.10	130.91	0	483	0.00	3.69	
12	4.90	128.95	1	422	0.20	3.27	
13	3.20	111.97	0	149	0.00	1.33	
14	3.00	104.35	0	213	0.00	2.04	
15	4.90	97.63	0	238	0.00	2.44	
16	4.00	86.35	0	322	0.00	3.73	
17	3.60	77.60	2	476	0.56	6.13	
18	3.60	74.48	1	109	0.28	1.46	
19	4.20	54.93	1	519	0.24	9.45	
20	3.80	59.23	1	215	0.26	3.63	
21	3.40	44.73	0	53	0.00	1.18	
22	5.70	45.60	4	152	0.70	3.33	

* Paired *t* test between DMCs density in centromere and DMCs density in other regions. Mb, million base pair.

**FIGURE 2:**
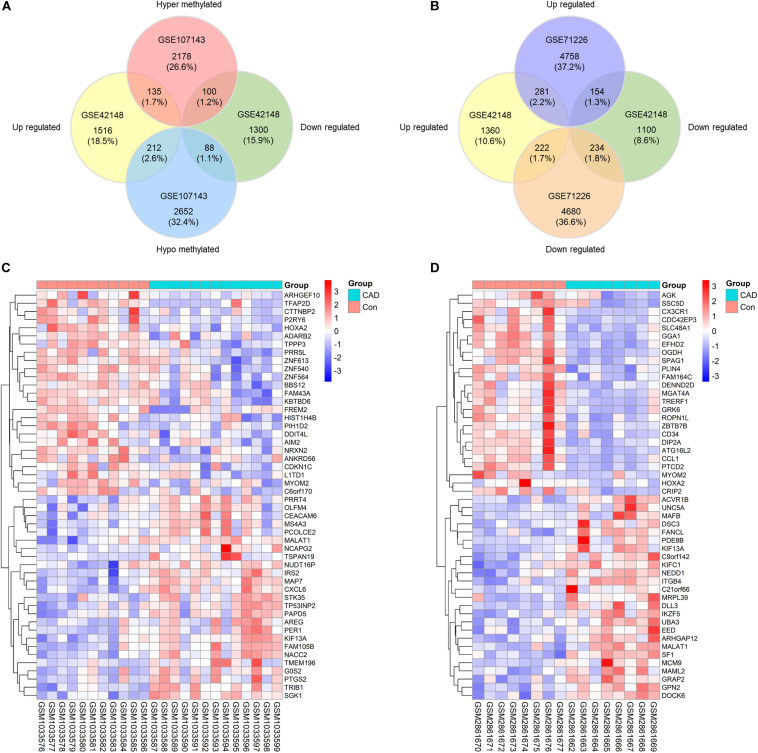
Identification of differentially expressed-methylated genes. **(A)** Venn plot of differentially expressed genes in dataset GSE42148 and differentially methylated genes in dataset GSE107143. **(B)** Venn plot of differentially expressed genes in dataset GSE42148 and GSE71226. **(C,D)** Representative heat map of the top 50 DEMGs in dataset GSE42148 and GSE107143.

To probe into the potential effect of the whole intragenic regions’ methylation status on gene function, we considered genes with one or more DMRs that differentially methylated in the same direction as DMGs. About 2413 hypermethylated genes and 2952 hypomethylated genes were classified based on DMRs. Subsequently, 135 genes were identified as up-regulated and hypermethylated (up-hyper genes), 212 genes were confirmed as up-regulated and hypomethylated (up-hypo genes), 100 genes were taken as down-regulated and hypermethylated (down-hyper genes), 88 genes were considered as down-regulated and hypomethylated (down-hypo genes) by overlapping DEGs and DMGs (Figure 2A).

Our integration analysis combined both expression and methylation data. The sensitivity and specificity of identifying potential functional genes might be affected when compared with traditional one type microarray data mining. Another expression microarray GSE71226 was analyzed as a quality assessment. As displayed in Figure 2B, there were 281 (2.2%) overlapped up-regulated genes, and 234 (1.8%) overlapped down-regulated genes in overlaps of GSE42148 and GSE71226 expression arrays. By contrast, a combination of expression and methylation arrays identified overlapped DEMGs with 135 (1.7%) up-hyper genes and 88 (1.1%) down-hypo genes (Figure 2A).

In total, 535 differentially expressed and methylated genes were screened out as DEMGs. It was worth noting that up-hyper genes and down-hypo genes occupied virtually half of DEMGs, which indicated the multidirectional regulation of methylation on gene function that is worth further study. Heatmaps were formed according to the hierarchical clustering of gene expressions or methylations levels to exhibit the top 50 ranked DEMGs by log_2_FC, respectively (Figures 2C,D). The full list of DEGs, DMCs, DMGs, and DEMGs can be found in Supplementary Table S2.

### Distributions of DMCs in Intragenic Regions

**FIGURE 3:**
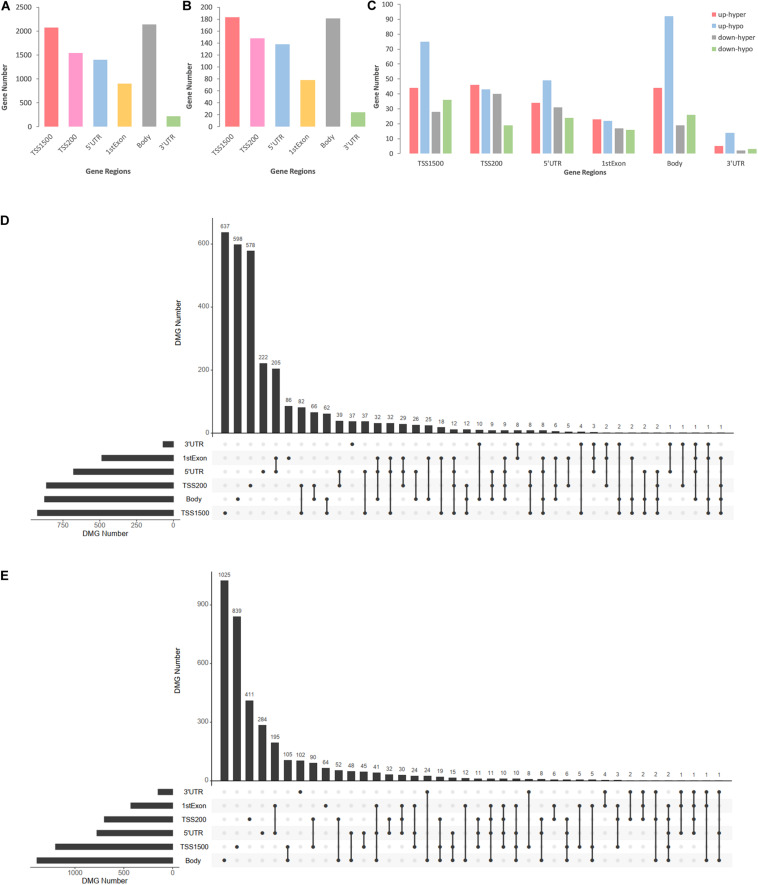
Consolidation analysis results of gene region distributions of intragenic DMCs. **(A)** Bar plot for intragenic DMCs in different regions of DMGs. **(B)** Bar plot for intragenic DMCs in different regions of DEMGs. **(C)** Bar plot for intragenic DMCs in different regions of four DEMGs groups. **(D)** UpSet plot for intragenic DMCs in different regions of hypermethylated genes. **(E)** UpSet plot for intragenic DMCs in different regions of hypomethylated genes.

DMCs were inhomogenously distributed in 6 intragenic regions of DMGs and DEMGs. As Figure 3A,B show, DMCs located in TSS1500 and the body of both DMGs and DEMGs accounted for over 50% of the total DMCs numbers. In contrast, there was less than 3% DMCs distributed in 3′UTR. In terms of linear lengths, the TSS1500 body is much longer than TSS200 and 5′UTR, and it is more reasonable to take TSS200 and 5′UTR as the high enrichment areas of DMCs. These results indicated the significant correlation of TSS200 and 5′UTR methylation status and gene expression. A similar distribution mode can be found in 4 kinds of DEMGs (Figure 3C). It can be observed that DMCs in 6 intragenic regions were mostly possessed by up-hypo genes, which manifested up-hypo genes as major roles in epigenetic regulation of CAD. These results indicate a significant correlation between TSS200 and 5′UTR methylation status and gene expression.

To demonstrate relevance among intragenic regions, UpSet plots were drawn to describe the methylation status of a certain DMGs with one or more DMRs. Over 70% of both hypermethylated genes and hypomethylated genes were single region-specific in TSS1500, TSS200, 5′UTR, or body (Figures 3D,E), while DMGs with multiple DMRs were mainly occupied by 5′UTR and 1stExon, TSS1500 and TSS200, TSS200, and 5′UTR. Even more noteworthy is the fact that approximately 6% of DMGs had 3 or more differentially methylated regions, which represented a general differential methylation status of a certain gene in CAD patients compared to controls.

### GO Functional and KEGG Pathway Enrichment Analysis of DEMGs

We performed GO functional and KEGG pathway enrichment analysis on up-hyper, up-hypo, down-hyper, down-hypo genes separately to explore the inner connection of DEMGs. The top 5 GO enrichment terms were illustrated in Table 3, from which we could find DEMGs enriched in numerous processes. Up-hyper genes were enriched in the biological process and 2 terms were associated with actin cytoskeleton reorganization. Four-fifths of terms enriched on up-hypo genes were related to organelle membrane or granule membrane. Notably, the rest 1 term of up-hypo genes was neutrophil activation, which enriched most genes among the top 5 terms. AS for down-hyper genes, GO terms were majorly centered on DNA-directed RNA polymerase activity, which indicates a potential connection between DNA methylation and mRNA expression. Besides, 2 terms of down-hypo genes were involved in the regulation of calcium ion transportation.

**TABLE 3:** List of top enriched GO terms of 4 DEMGs groups.

Category	Terms	ID	Description	Gene count	%	*P* value	Genes
Up-hyper genes	BP	GO:0034446	Substrate adhesion-dependent cell spreading	6	5.77	2.02E-05	ABL1, CDC42, FER, FN1, MICALL2, TRIOBP
	BP	GO:2000251	Positive regulation of actin cytoskeleton reorganization	3	2.88	1.52E-04	ABL1, BAIAP2L1, CDC42
	BP	GO:0042749	Regulation of circadian sleep/wake cycle	3	2.88	2.08E-04	CHRNB2, NR1D1, PER3
	BP	GO:0031532	Actin cytoskeleton reorganization	5	4.81	2.52E-04	ABL1, BAIAP2L1, CDC42, FER, MICALL2
	BP	GO:2000637	Positive regulation of gene silencing by miRNA	3	2.88	2.74E-04	DDX5, STAT3, TRIM71
Up-hypo genes	CC	GO:0016323	Basolateral plasma membrane	11	6.01	6.06E-06	ABCC1, AQP9, ATP6V1B1, B4GALT1, CD34, EZR, FLOT2, KCNQ1, MAP7, SLC19A1, SLC23A1
	CC	GO:0035579	Specific granule membrane	7	3.83	2.22E-05	CD59, MMP25, MS4A3, PLAUR, PLD1, PTPRJ, VAMP1
	CC	GO:0030667	Secretory granule membrane	12	6.56	2.33E-05	B4GALT1, CD59, CEACAM6, FLOT2, ICA1, MMP25, MS4A3, PLAUR, PLD1, PTPRJ, SLC11A1, VAMP1
	CC	GO:0033116	Endoplasmic reticulum Golgi intermediate compartment membrane	6	3.28	5.11E-05	AREG, CD59, CSNK1D, ERGIC1, MPPE1, NAT8
	BP	GO:0042119	Neutrophil activation	15	8.62	6.84E-05	B4GALT1, CAP1, CD59, CEACAM6, CXCL6, IMPDH1, MMP25, MMP9, MPO, MS4A3, OLFM4, PLAUR, PLD1, PTPRJ, SLC11A1
Down-hyper genes	MF	GO:0003899	DNA-directed 5′-3′ RNA polymerase activity	3	3.53	9.99E-04	CD3EAP, POLR3A, ZNRD1
	CC	GO:0030008	TRAPP complex	2	2.30	1.23E-03	TRAPPC4, TRAPPC5
	CC	GO:0055029	Nuclear DNA-directed RNA polymerase complex	4	4.60	1.29E-03	CD3EAP, POLR3A, RPRD1A, ZNRD1
	MF	GO:0034062	5′-3′ RNA polymerase activity	3	3.53	1.31E-03	CD3EAP, POLR3A, ZNRD1
	MF	GO:0097747	RNA polymerase activity	3	3.53	1.31E-03	CD3EAP, POLR3A, ZNRD1
Down-hypo genes	BP	GO:0034644	Cellular response to UV	4	5.80	2.40E-04	CRIP1, DHX36, TREX1, TRIAP1
	BP	GO:0002244	Hematopoietic progenitor cell differentiation	5	7.25	3.71E-04	C12orf29, DHX36, TCF12, TREX1, ZBTB24
	BP	GO:0051281	Positive regulation of release of sequestered calcium ion into cytosol	3	4.35	4.33E-04	F2RL3, P2RY6, TRPC1
	BP	GO:0045668	Negative regulation of osteoblast differentiation	3	4.35	8.86E-04	HOXA2, ID3, LIMD1
	BP	GO:0010524	Positive regulation of calcium ion transport into cytosol	3	4.35	1.05E-03	F2RL3, P2RY6, TRPC1

Table 4 exhibits top KEGG pathways of 4 kinds DEMGs. Enrichment analysis suggested up-hyper genes were significantly enriched in the VEGF signaling pathway and adipocytokine signaling pathway that might link with blood lipids. Up-hypo genes are mainly enriched in autophagy, vitamin digestion and absorption, and PI3K-Akt signaling pathway. There were fewer KEGG pathways enriched in down-hyper and down-hypo genes by comparison with up-regulated DEMGs. Only RNA polymerase and Fanconi anemia pathways, were identified in down-hyper genes. Down-hypo genes were associated with other types of O-glycan biosynthesis, cytosolic DNA-sensing pathway, mRNA surveillance pathway, and non-homologous end-joining.

**TABLE 4:** List of top enriched KEGG terms of 4 DEMGs groups.

Category	ID	Description	Gene count	%	*P* value	Genes
Up-hyper genes	hsa04370	VEGF signaling pathway	5	9.80	3.44E-05	AKT2, CDC42, KDR, PPP3CA, PTGS2
	hsa05206	MicroRNAs in cancer	8	15.69	7.21E-04	ABL1, IRS2, MARCKS, PTGS2, RPS6KA5, SOX4, STAT3, TRIM71
	hsa04722	Neurotrophin signaling pathway	5	9.80	9.38E-04	ABL1, AKT2, CDC42, IRAK3, RPS6KA5
	hsa04920	Adipocytokine signaling pathway	4	7.84	9.57E-04	AKT2, IRS2, RXRG, STAT3
	hsa05165	Human papillomavirus infection	8	15.69	1.08E-03	AKT2, CDC42, FN1, ITGB4, MAML2, MAML3, PTGS2, THBS4
Up-hypo genes	hsa04140	Autophagy - animal	7	7.87	8.25E-04	ATG16L2, CAMKK2, CFLAR, KRAS, PPP2CA, PTEN, TP53INP2
	hsa04977	Vitamin digestion and absorption	3	3.37	2.32E-03	ABCC1, SLC19A1, SLC23A1
	hsa04710	Circadian rhythm	3	3.37	4.87E-03	BHLHE40, CSNK1D, PER1
	hsa04151	PI3K-Akt signaling pathway	10	11.24	6.00E-03	AREG, CSF3R, IFNAR2, KIT, KRAS, MYB, PIK3R6, PPP2CA, PTEN, SGK1
	hsa05221	Acute myeloid leukemia	4	4.49	6.59E-03	KIT, KRAS, MPO, RUNX1
Down-hyper genes	hsa03020	RNA polymerase	2	7.41	4.87E-03	POLR3A, ZNRD1
	hsa03460	Fanconi anemia pathway	2	7.41	1.43E-02	FANCF, FANCL
Down-hypo genes	hsa00514	Other types of O-glycan biosynthesis	2	6.90	1.21E-02	GALNT3, LFNG
	hsa04623	Cytosolic DNA-sensing pathway	2	6.90	2.19E-02	AIM2, TREX1
	hsa03015	mRNA surveillance pathway	2	6.90	4.31E-02	PPP2R2B, RNGTT
	hsa03450	Non-homologous end-joining	1	3.45	4.65E-02	RAD50

### PPI Network Establishment, a Module Analysis, and Hub Genes Identification

To excavate the interaction among DEMGs, PPI networks based on STRING were visualized by Cytoscape for 4 kinds of DEMGs, respectively. The radius of each gene circle in the PPI network was positively associated with the absolute value of log_2_FC of mRNA expression between CAD patients and controls. Analogously, the color depth of each gene circle represents the methylation status of genes in the PPI network. The darker the color, the greater the difference of methylation levels between 2 study populations. Module analysis was performed to simplify the PPI network and focus on certain modules with stronger interactions. Subsequently, hub genes were filtered by 12 algorithms based on the bioinformatics analysis.

**FIGURE 4:**
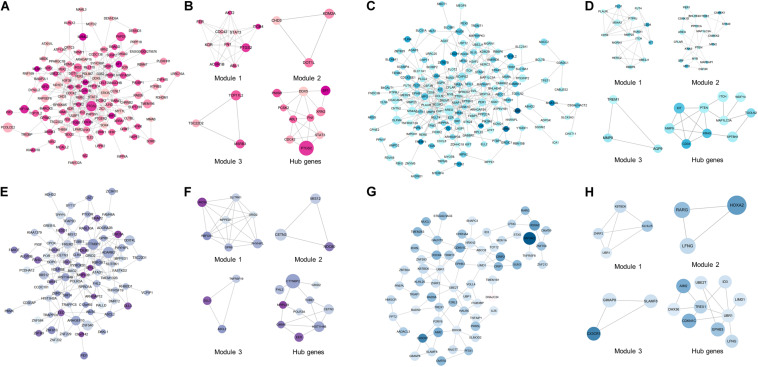
PPI network, top 3 modules, and top 10 hub genes of 4 DEMGs groups. **(A,B)** Up-hyper genes. **(C,D)** Up-hypo genes. **(E,F)** Down-hyper genes. **(G,H)** Down-hypo genes.

As displayed in Figure 4A,B, the top 10 hub genes of up-hyper genes are FN1, STAT3, CDC42, DDX5, ABL1, PTGS2, PCSK2, PNISR, XRN2, and SF1. PPI network, top 3 modules, and top 10 up-hypo hub genes were shown in Figures 4C,D. More precisely, the top 10 hub genes of up-hypo genes including PTEN, KRAS, MMP9, MAP1LC3A, ITCH, TGOLN2, CD34, KIT, WDFY4, and SPTBN1. The same module analysis and hub gene screening were performed on down-hyper genes, identifying the top 10 hub genes were POLR3A, HIST1H4B, CTTNBP2, EED, CETN3, TUBE1, FHL2, GRID2, GMNN, and MRPL39 (Figures 4E,F). While the top 10 hub genes based on interaction information from the PPI network of down-hypo genes were UBR1, TREX1, CDKN1C, UBE2T, ID3, DHX36, LFNG, LIMD1, EPHB3, and AIM2 (Figures 4G,H).

### GO Functional Enrichment Analysis of Hub Genes

**FIGURE 5:**
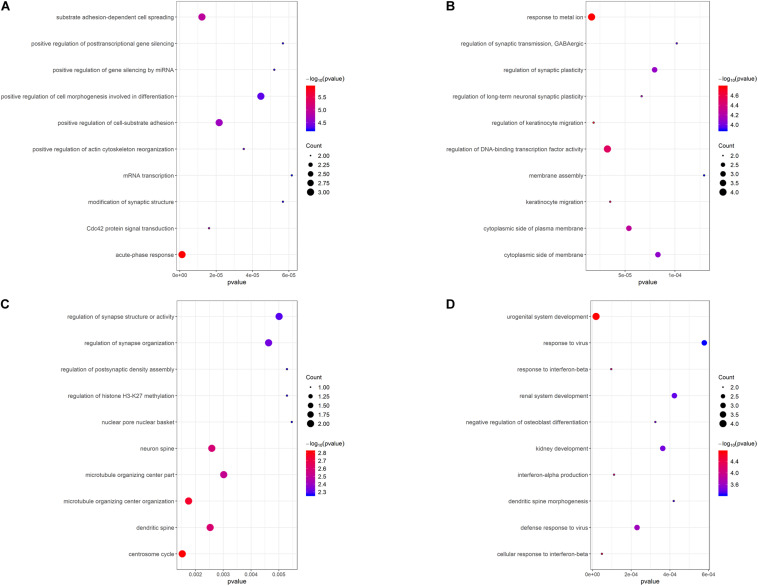
GO functional enrichment analysis of the top 10 hub genes of 4 DEMGs groups. **(A)** Up-hyper hub genes. **(B)** Up-hypo hub genes. **(C)** Down-hyper hub genes. **(D)** Down-hypo hub genes.

To illustrate the functional interrelation of hub genes, GO enrichment analysis was conducted in the top 10 hub genes of 4 DEMGs subgroups (Figure 5). Up-hyper hub genes were majorly enriched in terms of acute-phase response and positive regulation of the post-transcription. Regulation of synaptic function and membrane biogenesis were the main terms enriched in up-hypo genes. Intriguingly, down-hyper genes were observed to be associated with the regulation of histone H3-K27 methylation and participated in the regulation of postsynaptic in ways that were similar to up-hypo genes. Terms enriched in down-hypo genes were mostly related to response to interferons and urogenital system development.

### Clinical Characteristics of Subjects in the Validation Study

The clinical parameters of CAD patients and controls are summarized in Table 1. Groups of participants were matched in terms of gender and age. More CAD patients suffered from hypertension (HP) (*P* = 0.009) and diabetes mellitus (DM) (*P* = 0.025) compared with the controls. Among the serum lipid parameters, no conspicuous differences were observed in total cholesterol (TC), low density lipoprotein cholesterol (LDL-C), high density lipoprotein cholesterol (HDL-C). While the level of triglyceride (TG) was much higher in CAD patients (*P* < 0.001), the leucocyte differential count revealed a significant increase of monocytes (*P* < 0.001) and neutrophils (*P* = 0.031), but a prominent decrease of lymphocytes (*P* = 0.025) in CAD patients. Parameters based on leucocyte differential counts such as the lymphocyte to monocyte ratio (LMR) (*P* < 0.001) were decreased while the neutrophil to lymphocyte ratio (NLR) (*P* < 0.001) was increased in CAD patients.

### Hub Genes’ Expression and Methylation Status in the Validation Study

**FIGURE 6:**
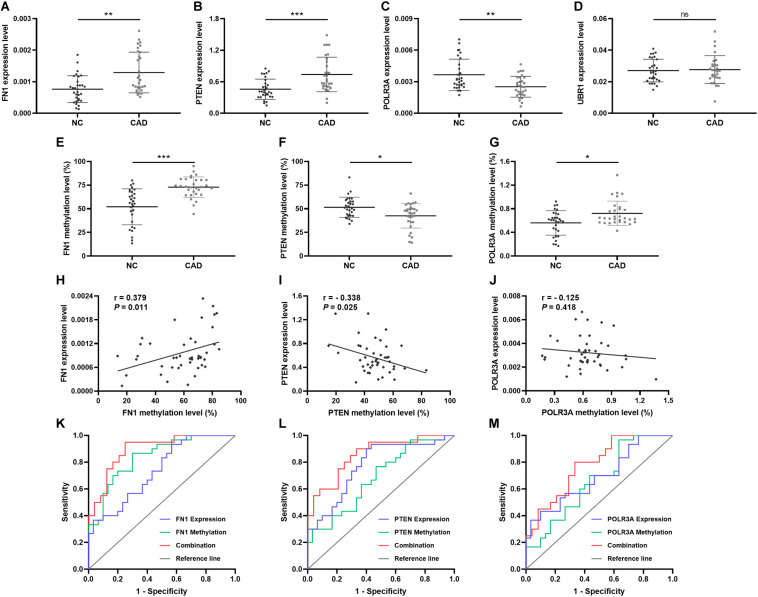
Relative expression, methylation, and ROC of top hub genes from 4 DEMGs subgroups. Expression level in 30 CAD patients and 30 controls. **(A)** FN1. **(B)** PTEN. **(C)** POLR3A. **(D)** UBR1. Methylation levels in 30 CAD patients and 30 controls. **(E)** FN1. **(F)** PTEN. **(G)** POLR3A. Correlation between expression and methylation. **(H)** FN1. **(I)** PTEN. **(J)** POLR3A. ROC is based on expression and methylation separately or combined. **(K)** FN1. **(L)** PTEN. **(M)** POLR3A. All experiments were performed twice. *P < 0.05, **P < 0.01, ***P < 0.001.

In order to validate the significance of the hub genes identified during bioinformatics analysis, the mRNA expression levels of top 1 hub genes from 4 DEMGs groups were detected by qPCR. In accord with the bioinformatics results, the FN1 (*P* = 0.001) from up-hyper genes and PTEN (*P* < 0.001) belonged to up-hypo genes and were remarkably upregulated in CAD patients when compared with controls (Figures 6A,B). The mRNA expression level of POLR3A, the top 1 hub gene of down-hyper genes, was conspicuously decreased in patients with CAD, which was also consistent with the bioinformatics results (*P* = 0.004, Figure 6C). Nevertheless, the foremost hub gene UBR1 from down-hypo genes was not differentially expressed between CAD patients and controls (*P* = 0.687, Figure 6D).

When considering the hub genes from DEMGs, we speculated that the significant expression differences of FN1, PTEN, and POLR3A were correlated with the aberrant DNA methylation status in CAD patients. According to the results of our bioinformatics analysis, FN1 was hypermethylated in 5′UTR, PTEN was hypomethylated in 5′UTR, and POLR3A was hypermethylated in TSS200. We detected the methylation status of corresponding regions by MDRE-qPCR and MSRE-qPCR. As displayed in Figure 6E, the 5′UTR of FN1 was prominently hypermethylated in CAD patients (*P* < 0.001). The methylation level was lower in the 5′UTR of PTEN in patients with CAD (*P* = 0.014, Figure 6F). TSS200 of POLR3A showed higher methylation status in CAD patients (*P* = 0.031, Figure 6G).

Meanwhile, the methylation of FN1 was positively related with the expression level (*r* = 0.379, *P* = 0.011, Figure 6H). Negative correlation between PTEN methylation and expression was demonstrated in Figure 6I (*r* = −0.338, *P* = 0.025). However, no obvious correlation was found in the methylation and expression of POLR3A (*r* = −0.125, *P* = 0.418, Figure 6J).

### Predictive Validity of Hub Genes

To evaluate the predictive validity of hub genes as potential biomarkers in CAD, ROC analysis was performed based on the mRNA expression and DNA methylation levels of hub genes separately or combinedly. As displayed in Figure 6K and Table 5, the expression (AUC = 0.738, *P* = 0.002) and methylation (AUC = 0.839, *P* < 0.001) of FN1 were with moderate diagnostic value. The predictive validity of FN1 was improved with the combination of expression and methylation data (AUC = 0.894, *P* < 0.001). Moderate diagnostic value was verified in the expression of PTEN (AUC = 0.776, *P* < 0.001, Figure 6L). Although the methylation of PTEN had lower predictive value (AUC = 0.683, *P* = 0.015), the predictive value was remarkably increased when combined with the expression data (AUC = 0.856, *P* < 0.001). POLR3A showed comparatively lower diagnostic validity in both expression (AUC = 0.691, *P* = 0.011) and methylation levels (AUC = 0.662, *P* = 0.031, Figure 6M). The diagnostic ability of POLR3A was improved by taking expression and methylation data into integration (AUC = 0.779, *P* = 0.002).

**TABLE 5:** ROC analysis based on the expression and methylation status of hub genes.

Hub gene	Classification	AUC	95% CI	*P* value	Se (%)	Sp (%)
FN1	Expression	0.738	0.614–0.861	0.002	93.333	43.333
	Methylation	0.839	0.740–0.938	<0.001	86.667	70.000
	Combination	0.894	0.801–0.987	<0.001	95.000	75.000
PTEN	Expression	0.776	0.656–0.895	<0.001	93.333	56.667
	Methylation	0.683	0.549–0.817	0.015	76.667	53.333
	Combination	0.856	0.746–0.967	<0.001	90.000	66.667
POLR3A	Expression	0.691	0.558–0.824	0.011	36.667	96.667
	Methylation	0.662	0.525–0.800	0.031	96.667	36.667
	Combination	0.779	0.645–0.913	0.002	80.000	66.667

*AUC, area under the receiver operating characteristic curves; Se, sensitivity; Sp, specificity.*

### Correlations Between Hub Genes Expressions and Clinical Characteristics

Correlation analysis and multivariate stepwise linear regression analysis were performed to investigate the underlying connection between the clinical characteristics of all enrolled subjects and expression levels of hub genes. As displayed in Table 6, both FN1 (*r* = 0.268, *P* = 0.039) and PTEN (*r* = 0.326, *P* = 0.011) were identified as positively correlated with monocyte counts. LMR was observed to be negatively related with FN1 (*r* = −0.255, *P* = 0.049) and PTEN (*r* = −0.315, *P* = 0.014), and positively correlated with POLR3A (*r* = 0.288, *P* = 0.026). Besides, PTEN was reversely associated with NMR (*r* = −0.311, *P* = 0.016) and the expression of POLR3A decreased with aging (*r* = −0.320, *P* = 0.013). In addition, although the expression of UBR1 was not confirmed to be different between CAD patients and controls, a prominent reverse correlation was verified between UBR1 and TG when all subjects were involved (*r* = −0.312, *P* = 0.012).

**TABLE 6:** Correlation analysis and multivariate stepwise linear regression analysis of gene expression with clinical parameters of all participants.

Characteristics	FN1 (*n* = 60)				PTEN (*n* = 60)				POLR3A (*n* = 60)				UBR1 (*n* = 60)			
	*r*	*P* value	β	*P* value	r	*P* value	β	*P* value	r	*P* value	β	*P* value	r	*P* value	β	*P* value
**Age**	0.221	0.090			0.134	0.308			−**0.320**	**0.013**	−**0.320**	**0.013**	−0.223	0.087		
Gender	−0.114	0.385			−0.087	0.508			0.030	0.817			0.099	0.449		
History of HP	0.083	0.530			0.141	0.284			−0.219	0.093			−0.140	0.285		
History of DM	0.151	0.250			0.019	0.884			0.050	0.707			−0.143	0.276		
TC	0.092	0.494			0.037	0.784			0.124	0.358			0.164	0.224		
**TG**	0.192	0.153			0.123	0.361			−0.071	0.598			−**0.312**	**0.012**	−**0.312**	**0.012**
LDL-C	0.052	0.701			0.014	0.918			0.145	0.283			0.132	0.327		
HDL-C	−0.129	0.341			−0.150	0.267			0.042	0.759			0.194	0.148		
WBC	−0.086	0.512			−0.066	0.619			−0.021	0.871			−0.030	0.818		
**Monocyte**	**0.268**	**0.039**	**0.268**	**0.039**	**0.326**	**0.011**	**0.326**	**0.011**	−0.089	0.497			0.022	0.866		
Neutrophil	0.004	0.974			0.070	0.593			−0.102	0.439			−0.102	0.440		
Lymphocyte	0.144	0.271			0.089	0.500			0.207	0.113			0.113	0.390		
**LMR**	−**0.255**	**0.049**	−0.216	0.055	−**0.315**	**0.014**	−0.203	0.086	**0.288**	**0.026**	0.104	0.128	0.075	0.568		
**NMR**	−0.125	0.342			−**0.311**	**0.016**	−0.235	0.069	0.046	0.730			−0.136	0.301		
NLR	0.230	0.077			0.025	0.847			−0.185	0.157			−0.120	0.361		

*Entries in bold font indicate statistically significant (*P* < 0.05).*

After adjustment of LMR by multivariate stepwise linear regression, there still existed a positive correlation of FN1 with monocyte counts (β = 0.268, *P* = 0.039). When eliminated the interference of LMR and NMR, PTEN was still associated with monocyte amounts (β = 0.326, *P* = 0.011), while POLR3A remained related to age (β = −0.320, *P* = 0.013) after adjusting LMR.

## Discussion

Identify epigenetic regulation patterns and certain biomarkers from PBLs would be conducive to the diagnosis, therapy, and monitor of CAD in a non-invasive approach. In this study, we filtrated genes that were both discrepantly expressed and methylated in CAD patients compared with controls. Pathways enriched by these genes were demonstrated and hub genes were screened out based on the PPI network. To verify the results of bioinformatics data analysis, expression and methylation levels of top hub genes were experimentally compared in CAD patients and controls. Furthermore, we investigated methylation patterns of different gene regions and gave evidence of the most vulnerable region to methylation in CAD. The differential expressions of top hub genes filtered from DEMGs were associated with altered DNA methylation status, which shed light on the underlying regulatory mechanism of DNA methylation in CAD and helped identify the novel nucleic acid biomarkers.

As the chromosome distribution map exhibited, differentially methylated CpGs covered almost every region of each chromosome. This can be regarded as the universality of methylated regulation in the pathogenesis of CAD (Deng et al., 2018). However, no DMCs were observed on sex chromosomes in our study, and a similar phenomenon was also observed in another published study of Parkinson’s disease (Wang et al., 2019). We speculated this was partly due to the relatively short liner length and fewer CpG sites on sex chromosomes. Meanwhile, it is interesting to note that DMCs distributed in regions near centromeres were relatively fewer than other regions. The phenomenon might be partly attributed to the supercoiling structure and hyper-reiterated DNA sequence around the centromeres (Ichikawa et al., 2017).

Numerous published studies took promoters as key differentially methylated regions in CAD, while other intragenic regions were less concerned (Heidari et al., 2019; Indumathi et al., 2019). Recently, a few studies have demonstrated methylation sites in the gene body or TSS1500 were also essential to the pathogenesis of CAD (Liu et al., 2017). Our research confirmed that the gene body and TSS1500 possessed almost half of DMCs, no matter in DMGs or DEMGs. Nevertheless, TSS200 and 5′UTR were the most enriched intragenic areas of DMCs when we considered the linear length. A portion of hypermethylation and hypomethylation genes showed discrepant methylation status in both 5′UTR and 1stExon, or TSS1500 and TSS200, or TSS200 and 5′UTR. Given the adjacent spatial positions of the 3 pairs regions, a rational inference could be raised that the methylation status of CpGs was spatiality and regionality. We divided DEMGs into 4 groups and demonstrated up-hypo genes as the major part functioning in the progress of CAD.

Contrary to the conventional concept that DNA methylation always negatively regulates gene expression, our study found that up-hyper genes and down-hypo genes made up 42% of DEMGs. The up-hyper and down-hypo genes have been reported in a few published studies. About 43% of 32 prognostic genes showed a significant positive correlation between the expression level and the DNA methylation status in breast cancer (Gyõrffy et al., 2016). In another report of lung cancer, the correlations between DNA methylation and gene expression were detected for approximately 750 genes, but for one-third of these, the correlations were positive (Bjaanæs et al., 2016). More than 30% of significant methylation-expression correlations were positive in human monocytes (Liu et al., 2013), however, no convincing explanation for this has yet been raised. We speculate that the aberrant methylated regions might encompass potential *cis-*acting elements, such as enhancers and silencers. The interaction between *cis-*acting elements and *trans-*acting elements can be multidirectional, depending on their characters in gene expression regulations. The regulatory mechanisms will become more complicated when the *cis-*acting elements have a different methylation status, given that methylation may influence the combining capacity of transcription factors through the changes of DNA spatial structure. These findings and hypotheses suggest a great diversity and complexity of epigenetic regulatory mechanisms and highlight the need for further molecular investigations.

Kyoto Encyclopedia of Genes and Genomes pathway enrichment analysis suggested up-hyper genes mainly enriched in adipocytokine signaling pathway and VEGF signaling pathway. This was consistent with previous studies in which increased expression of the adipocytokine omentin was detected in the epicardial adipose tissue of CAD patients and the unbalance of isoforms of VEGF was associated with the complexity and severity of CAD (Harada et al., 2016; Shibata et al., 2018). Up-hypo genes are primarily enriched in neutrophil activation and it has been confirmed in several studies that an increased neutrophil count was connected with the severity of CAD (Li et al., 2018). Intriguingly, hub genes belonged to down-hyper genes and were directly correlated with the regulation of histone H3-K27 methylation according to GO enrichment analysis, which indicated an underlying association between DNA methylation and histone methylation. Regulation of calcium ion transport into the cytosol was the term enriched by down-hypo genes and has been proved to affect the progress of CAD through the coronary smooth muscle (Badin et al., 2018).

Protein-protein interaction networks were established to excavate the interaction among DEMGs and filtrate the most central 10 hub genes from each group. Although these top hub genes were only enriched in several items from GO enrichment analysis, many of these enriched items were validated by published large-scale transcriptomics sequencing and DNA methylation studies in CAD. For instance, dysregulation of cell-substrate adhesion has been demonstrated to accelerate the progress of CAD by promoting macrophages migration in both peripheral blood and aortic tissue, which was accordant with our findings in up-hyper hub genes (Sinnaeve et al., 2009; Rask-Andersen et al., 2016). We observed that up-hypo hub genes were enriched in response to metal ion, a response also found in both peripheral blood and adipose tissue from CAD patients (Ek et al., 2016; Vacca et al., 2016). Besides, activation of the immune system was the main item that down-hypo hub genes enriched in, and similar results were indicated in plaques from the internal mammary artery, coronary artery, and great saphenous vein (Elashoff et al., 2011; Nazarenko et al., 2015). These findings indicate the molecular mechanism intercommunity in various tissues when organisms suffer from CAD.

Because of the small sample size of datasets obtained from the GEO database and accumulative deviation from bioinformatics analysis, we performed laboratory verification with a considerable sample size for each top 1 hub genes in each DEMGs subgroup. The results suggested accordant expression and methylation levels of FN1, PTEN, and POLR3A in comparison with bioinformatics analysis results. The expression of FN1 was positively related to the methylation level. Negative relevance was found between the expression and methylation status of PTEN. These significant correlations hinted that aberrant DNA methylation was involved in the regulation of FN1 and PTEN expression in CAD patients. To further investigate the mechanism of FN1, PTEN, and POLR3A in the pathogenesis of CAD, correlation analysis and multivariate stepwise linear regression analysis were performed. FN1 was positively correlated with monocyte counts. A positive correlation was also observed in PTEN with monocyte amounts. POLR3A was negatively related to age. These results indicated that FN1 and PTEN might function in the system infection since the monocytosis was the acknowledged systemic infection index.

Inflammatory cytokines activated TGF-β signaling pathway, which promoted the expression of FN1 in human endothelial cells (Chen et al., 2015). Fibronectin, encoded by FN1, was enriched in vascular subendothelial basement membrane during the early process of atherosclerotic plaque formation and aggravated the monocytes recruitment (Al-Yafeai et al., 2018). The significant up-regulation of FN1 was positively related to monocyte amounts in CAD patients. In considering the significant correlation between the expression and 5′UTR methylation of FN1, it could be inferred that the aberrant 5′UTR methylation status of FN1 induced the over expression of FN1 and triggered the recruitment of monocytes in CAD patients. However, the methylation status of 5′UTR of FN1 was positively related to the expression level, which was opposite to the conventional concept that DNA methylation was always negatively correlated with gene expressions. Since the 5′UTR of FN1 was hypermethylated, the upstream of FN1 might also be hypermethylated. We speculated the upstream of FN1 encompassed silencers, which were incapacitated due to hypermethylation. Further studies are needed to verify the hypothesis. Vascular endothelial cell injury, caused by chronic inflammation in atherosclerosis, accelerated atherosclerotic plaque formation. A series of microRNAs could bind to the 3′UTR of PTEN, altering the proliferation of vascular endothelial cells through the PI3K-Akt pathway and influencing the procession of CAD (Wang et al., 2017). Previous reports have suggested that PTEN was up-regulated in the peripheral blood mononuclear cells of CAD patients, which was consistent with our findings in PBLs and confirmed the reliability of our research (Nariman-Saleh-Fam et al., 2019). Besides the involvement of microRNAs in the regulation of PTEN, we proved that the 5′UTR methylation of PTEN might also participate in the regulation network. Currently, no study was carried out to research the function of POLR3A in CAD or atherosclerosis. POLR3A was reported to mainly trigger leukodystrophy (Choquet et al., 2017). The correlation between POLR3A methylation and expression was not statistically significant, hinting there might be other elements that participated in the expression regulation of POLR3A, such as the wildly reported POLR3A mutations, miRNAs and transcript factors. The aberrant expression and methylation of POLR3A may indicate a new target for CAD, while the regulatory mechanisms still need further investigation.

In summary, our study consolidated both mRNA expression and DNA methylation microarrays of PBLs in CAD into bioinformatics analysis and executed experimental validation. The methylation patterns of CAD were profiled based on the distribution of DMCs in intragenic regions. FN1, PTEN, and POLR3A were screened as top hub genes through bioinformatics analysis and were confirmed through subsequent experimental verification in a Chinese case-control study. Further molecular and clinical experiments with a larger sample size are needed to illuminate the underlying mechanism of the differential expression and methylation of FN1, PTEN, and POLR3A in the pathogenesis of CAD.

## Pre-Print

This manuscript has been released as a pre-print at Research Square (Zhang XK, Xiang Y, He DD, et al.) (Zhang et al., 2020).

## Data Availability Statement

Gene Expression omnibus (GEO) was the source of the primary data. The gene expression data can be found at https://www.ncbi.nlm.nih.gov/geo/query/acc.cgi?acc=GSE42148 and https://www.ncbi.nlm.nih.gov/geo/query/acc.cgi?acc=GSE71226. DNA methylation data can be downloaded at https://www.ncbi.nlm.nih.gov/geo/query/acc.cgi?acc=GSE107143.

## Ethics Statement

The studies involving human participants were reviewed and approved by Medical Ethics Committee of Zhongnan Hospital of Wuhan University. Written informed consent for participation was not required for this study in accordance with the national legislation and the institutional requirements.

## Author Contributions

FZ conceived and designed the workflow. XZ performed the experiments and analyzed the data. XZ and FZ wrote the manuscript. YX collected the samples. DH analyzed the data and created the figures. BL, CW, and JL revised the manuscript. All authors approved the final manuscript.

## Conflict of Interest

The authors declare that the research was conducted in the absence of any commercial or financial relationships that could be construed as a potential conflict of interest.

## Supplementary Material

FIGURE S1 Flow diagram of the analysis process.

TABLE S1 Primer sequences and Tm for qPCR.

TABLE S2 Full list of DEGs, DMCs, DMGs, and DEMGs.
