# Supplementary figures and images for: Identification of Potential Biomarkers for CAD Using Integrated Expression and Methylation Data
Source: Front Genet. 2020 Sep 9;11:778. doi: 10.3389/fgene.2020.00778 (PMC7509170; doi:10.3389/fgene.2020.00778)

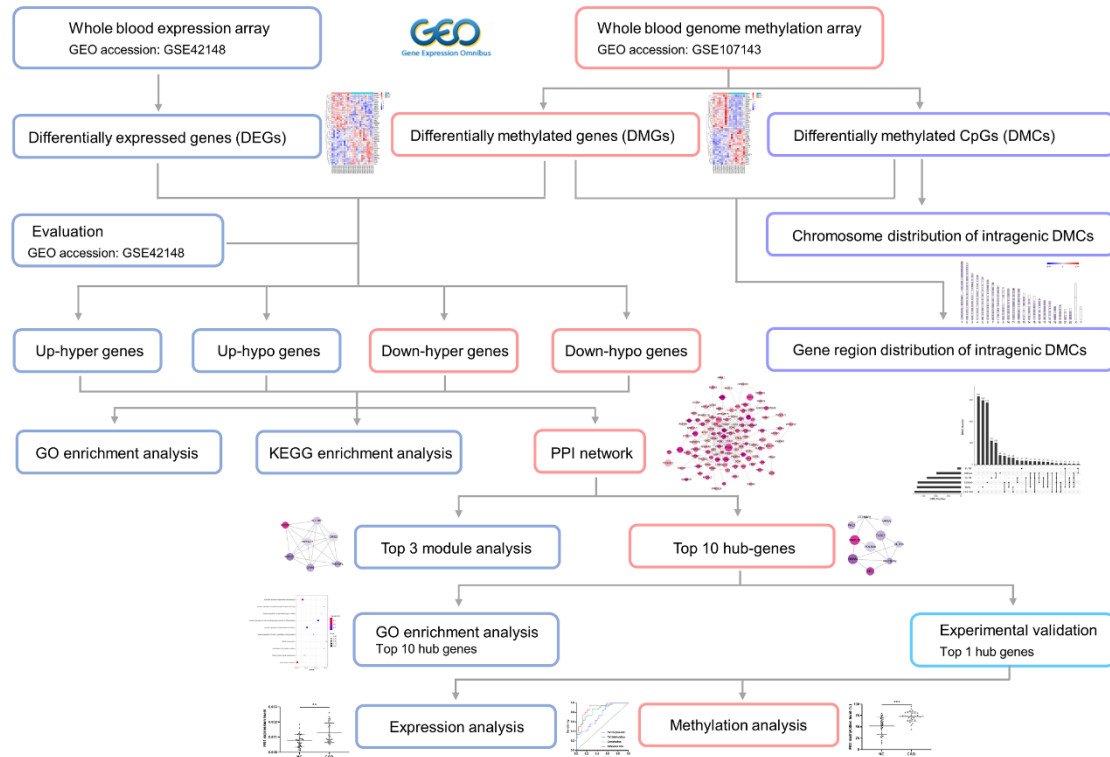

**Figure S1.** Flow diagram of the analysis process.

Supplement: FIGURE S1 — Flow diagram of the analysis process. [file Image_1.PDF]
