# Supplementary material for: Identification of Potential Biomarkers for CAD Using Integrated Expression and Methylation Data
Source: Front Genet. 2020 Sep 9;11:778. doi: 10.3389/fgene.2020.00778 (PMC7509170; doi:10.3389/fgene.2020.00778)
Supplement: TABLE S1 — Primer sequences and Tm for qPCR. [file Table_1.PDF]

Table S1. Primer sequence and Tm for qPCR.

Gene	Classification	Primer sequence（5'→3'）	Tm（℃）	Product length（bp）
FN1	Expression	F: ACAAGCATGTCTCTCTGCCAA	60	192
		R: GCAATGTGCAGCCCTCATTT		
	Methylation	F: CTAAGCATGTTGAGACGGTGG	60	195
		R: TTCTCTGGTCCTCTGCATCC		
PTEN	Expression	F: TTTGAAGACCATAACCCACCAC	60	134
		R: ATTACACCAGTTCGTCCCTTTC		
	Methylation	F: TCTCATCTCCCTCGCCTGA	60	159
		R: GTGATGTGGCGGGACTCT		
POLR3A	Expression	F: CCAAGTTGTGAGTAAGAACCTGT	60	81
		R: CCCATCCTATGGTCGAGCAC		
	Methylation	F: AGACTTCCGGCATGCCTTTT	60	210
		R: ATGCAAGTTTTGGCCCTCTG		
UBR1	Expression	F: TTTGTGGGAGGGTTTTCAAAAGT	60	186
		R: CAGTTTTCCATGCCTCTGTGT		
